# Retinoblastoma and Its Binding Partner MSI1 Control Imprinting in *Arabidopsis*

**DOI:** 10.1371/journal.pbio.0060194

**Published:** 2008-08-12

**Authors:** Pauline E Jullien, Assaf Mosquna, Mathieu Ingouff, Tadashi Sakata, Nir Ohad, Frédéric Berger

**Affiliations:** 1 Chromatin and Reproduction Group, Temasek Life Sciences Laboratory, National University of Singapore, Singapore, Republic of Singapore; 2 Zentrum für Molekularbiologie der Pflanzen (ZMBP), Entwicklungsgenetik, Universität Tübingen, Tübingen, Germany; 3 Department of Plant Sciences, Faculty of Life Sciences, Tel-Aviv University, Tel-Aviv, Israel; University of Bath, United Kingdom

## Abstract

Parental genomic imprinting causes preferential expression of one of the two parental alleles. In mammals, differential sex-dependent deposition of silencing DNA methylation marks during gametogenesis initiates a new cycle of imprinting. Parental genomic imprinting has been detected in plants and relies on DNA methylation by the methyltransferase MET1. However, in contrast to mammals, plant imprints are created by differential removal of silencing marks during gametogenesis. In *Arabidopsis*, DNA demethylation is mediated by the DNA glycosylase DEMETER (DME) causing activation of imprinted genes at the end of female gametogenesis. On the basis of genetic interactions, we show that in addition to DME, the plant homologs of the human Retinoblastoma (Rb) and its binding partner RbAp48 are required for the activation of the imprinted genes *FIS2* and *FWA*. This Rb-dependent activation is mediated by direct transcriptional repression of MET1 during female gametogenesis. We have thus identified a new mechanism required for imprinting establishment, outlining a new role for the Retinoblastoma pathway, which may be conserved in mammals.

## Author Summary

Imprinting in plants and mammals involves a process whereby one of the two inherited gene variants (alleles) is inactivated. During imprinting, the transcriptional silencing of one allele is mediated by histone modifications or DNA methylation. The expressed parental allele is activated during gametogenesis by poorly understood mechanisms that remove silencing marks. In *Arabidopsis*, we studied genes expressed only from the maternal allele because the paternal allele is silenced by DNA methylation. We report that the expression of the maternal allele requires the repression of transcription of the major DNA methyltransferase by the sustained activity of the *Arabidopsis* homologs of the Retinoblastoma pathway. Repression is confined to the female gamete and is essential for the expression of imprinted genes in plants. The conserved transcriptional repression of DNA methyltransferases by the Retinoblastoma pathway suggests that this new regulation of imprinting might be also active in mammals.

## Abstract 3

A new regulation of imprinting discovered in *Arabidopsis* involves the Retinoblastoma gene.

## Introduction

Genomic imprinting causes parental allele-specific expression during early development in mammals and plants [1]. In mammals, silencing marks are acquired by de novo DNA methylation during gametogenesis. Each imprinted locus receives a mark depending on its parental origin [2]. In embryonic cells, epigenetic marks are maintained on the silenced allele by a semiconservative mechanism involving the DNA methyltransferase Dnmt1 [3]. Following DNA replication, Dnmt1 methylates preferentially hemimethylated DNA, resulting in the maintenance of silencing marks on one parental allele. In the embryo, imprinting marks are read, leading to monoallelic gene expression. Certain genes remain imprinted and expressed in the adult [4]. A new imprinting cycle is initiated in the embryonic primordial germ cells where the epigenetic marks are lost following a global demethylation of DNA. The mechanism by which such demethylation originates is still unknown [5].

Embryogenesis in flowering plants and mammals depends on the function of embryo-nurturing annexes, the endosperm in plants and the placenta in mammals. In mammals, the placenta derives from the trophoblastic lineage separated from the embryonic lineage at the blastocyst stage. In flowering plants, the endosperm lineage separates from the embryonic lineage during female gametogenesis before fertilization [6]. The meiotic spore produces the haploid gametophyte in which two specialized female gametes differentiate: the egg cell and the central cell [7]. The fertilized egg cell produces the embryo, and the fertilized central cell generates the endosperm [8]. All imprinted genes known to date in plants are solely expressed in the endosperm, and some of these are essential for endosperm development [9].

Only a few imprinted genes have been identified in *Arabidopsis* [1]. *MEDEA* (*MEA*) [10], *FWA* [11], and *FERTILIZATION INDEPENDENT SEED 2* (*FIS2*) [12] are only expressed from the maternal allele, whereas *PHERES1* (*PHE1*) is preferentially expressed from the paternal allele [13,14]. *PHE1* and *FWA* encode transcription factors of unknown function in the endosperm. *MEA* and *FIS2* encode subunits of a Polycomb Group (Pc-G) complex [15]. Both alleles of *MEA*, *FIS2*, and *FWA* are silenced by distinct epigenetic mechanisms throughout the plant lifecycle until gametogenesis takes place. Silencing of *MEA* results from histone 3 lysine 27 (H3K27) trimethylation by Pc-G complexes [16,17], whereas silencing of *FWA* and *FIS2* is mediated by the DNA METHYLTRANSFERASE 1 (MET1), which maintains DNA methylation of CpG sites [11,12]. Silencing marks are maintained in the sperm cells during male gametogenesis. During endosperm development, the inherited paternal copy remains silenced by MET1 or Pc-G activities, whereas the maternal copy is inherited as transcriptionally active, which results in monoparental expression [1].

During female gametogenesis, the expression of *MEA*, *FIS2*, and *FWA* is activated specifically in the central cell [18,19]. The activation of the above genes relies on DEMETER (DME), a DNA glycosylase protein [11,12,19]. DME removes methylated cytosines [16,20], causing the loss of the silencing marks from *FIS2* and *FWA* promoters and allowing transcription in the central cell. The maternal allele inherited from the central cell remains active after fertilization in the endosperm while the paternal allele remains silenced. Hence the gene is imprinted in the endosperm while silenced in all other tissues. Loss of DNA methylation from the promoter of imprinted genes is conserved in the maize central cell [21,22]. Imprinting in plants thus results from the loss of silencing epigenetic marks during gametogenesis, which leads to gene expression. However, persisting expression of *FIS2* in the *dme* mutant [12] suggested that mechanisms parallel to DME lead to removal of DNA methylation marks from *FIS2* during female gametogenesis.

In mammalian cell cultures, the expression of the DNA methyltransferase Dnmt1 is controlled by the Retinoblastoma pathway [23–25]. The Retinoblastoma protein (pRb) is known to repress the expression of S-phase genes during the G1 phase of the cell cycle through inhibition of E2F transcription factors. The pRb binding protein RbAp48 is critical for this function [26]. The *Arabidopsis* pRb homolog RETINOBLASTOMA RELATED 1 (RBR1) is highly expressed in the mature central cell, preventing uncontrolled syncytial proliferation [27]. MULTICOPYSUPPRESSOR OF IRA1 (MSI1) protein, a RbAp48 homolog, prevents production of endosperm from unfertilized central cells [28,29]. However, the interaction between RBR1 and MSI1 was not shown in *Arabidopsis*. We hypothesized that in *Arabidopsis*, the Retinoblastoma pathway may control the expression of *MET1*. In this study, we demonstrate that *Arabidopsis* MSI1 and RBR1 interact in vivo and down-regulate *MET1* directly during female gametogenesis. Reduction of MET1 activity by the Retinoblastoma pathway in the central cell is essential for transcriptional activation of *FIS2* and *FWA.* We thus provide evidence for a new mechanism essential for the activation of MET1-dependent imprinted genes in plants.

## Results

### RBR1 Interacts with MSI1

**Figure 1:**
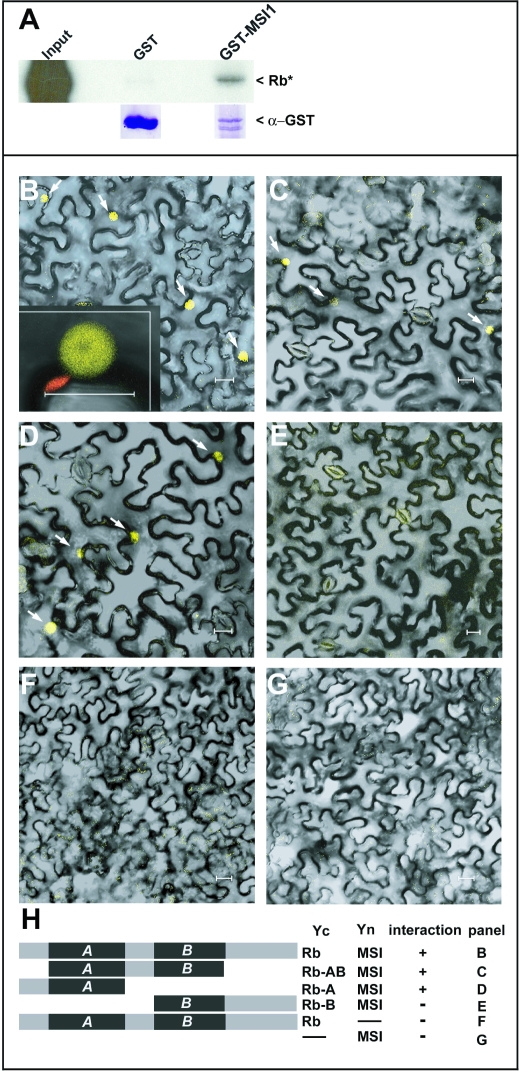
RBR1 Interacts with MSI1 (A) Pull-down assay testing for interaction between *Arabidopsis* MSI1 and RBR1. The full-length RBR1 protein, labeled with [^35^S] methionine, was incubated with GST-MSI1 protein bound to agarose beads (GST MSI). As a negative control, the labeled protein was incubated with GST only, bound to agarose beads (GST). Input indicates the labeled protein used in the binding assays. Immunodetection of GST and GST-MSI1 were performed using anti-GST antibody. (B–H) BiFC analyses showing in vivo interaction between RBR1 and MSI1 proteins. Fluorescence is observed in nuclei following YFP reconstitution between YN-MSI1 and YC-RBR1 (B) The inset represents the detail of an individual nucleus. In comparison, either YN with YC-RBR1 (F) or YN-MSI1 with YC (G) serving as negative controls display no fluorescence. (C–E) Truncation of the RBR1 protein showing that only the RB-A domain is required for the interaction with MSI1. Localization was determined in leaf epidermis of N. benthamiana. YFP fluorescence from single confocal sections showing a fraction of the nuclei from all cells in the field was overlaid with Nomarsky differential interference contrast (DIC) images. Arrows point to nuclei expressing YFP fluorescence. Scale bars represent 20 μm. (H) Representation of the different RBR1 truncations tested for interaction with MSI1 in the assays shown in (A) to (G).

Interaction between the tomato homolog of MSI1 and human or maize homologs of RBR1 has been shown in vitro [30]. Employing an in vitro glutathione S-transferase (GST) pull-down assay, we have detected direct interaction between *Arabidopsis* MSI1 and RBR1 proteins (Figure 1A). In order to test whether the *Arabidopsis* MSI1 and RBR1 proteins interact in living cells, we have used the bimolecular fluorescence complementation (BiFC) assay [31]. *MSI1* and *RBR1* coding sequences were fused to the sequences encoding the N-terminal (YN) or the C-terminal (YC) part of the yellow fluorescent protein (YFP). We observed YFP fluorescence from nuclei in which YN-MSI1 and YC-RBR1 were transiently cotransformed into leaf epidermis and reconstituted a functional YFP protein (Figure 1B). No fluorescence was observed using YN alone with YC-RBR1 or using YN-MSI1 with YC alone (Figure 1F and 1G). We thus concluded that MSI1 and RBR1 interact in vitro and in vivo as shown previously between homologs of RBR1 and MSI1 in human, *Drosophila*, and maize [32].

In order to identify which part of the RBR1 protein is required for the interaction with MSI1, we performed a deletion analysis. The *Arabidopsis* RBR1 protein contains the two conserved domains RB-A and RB-B (Figure 1H). Human Retinoblastoma RB-A and RB-B domains form a tridimensional structure called the A/B pocket. Human pRb interacts through the A/B pocket with the LxCxE domain of the histone deacetylase 1 (HDAC1) [33], which in turns binds RbAp48 [26,34]. Using truncations of the RBR1 protein fused to YC, we demonstrated that the interaction between MSI1 and RBR1 occurs through the RB-A domain (Figure 1B–1E). We concluded that the interaction between MSI1 and RBR1 takes place in absence of the A/B pocket, suggesting that an HDAC1 is not involved in this interaction. In addition, the maize HDAC ZmRpd3I [35] and all *Arabidopsis* HDAC homologs do not contain the LxCxE domain, which is required for the interaction between Rb, HDAC1, and RbAp48 in mammals. In conclusion, we suggest that in contrast to mammals, the plant homologs of Rb and RbAp48 likely interact directly and may not require an HDAC1 homolog.

### MSI1 Represses the Expression of the DNA Methyltransferase MET1

**Figure 2:**
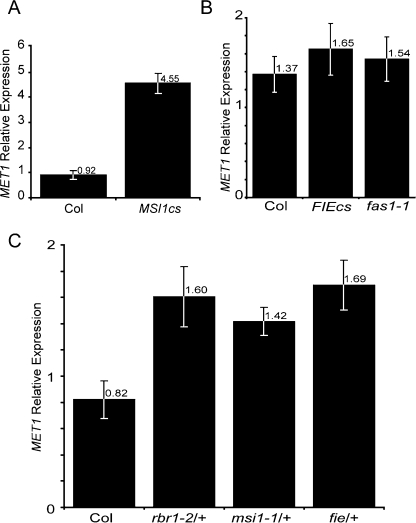
*MSI1* Represses *MET1* Expression (A) Q-PCR analyses on RNAs from mature leaves show an increase of *MET1* expression in *MSI1cs* in comparison to wild-type Columbia. The RQ value corresponds to the average of five independent biological replicates. (B) Q-PCR analyses on RNAs from *FIEcs* and *fas1* mature leaves. (C) Q-PCR analyses showing an increase of MET1 expression in *rbr1–1/+*, *msi1–1/+*, and *fie/+* ovules at 1.5 d after emasculation (DAE) in comparison to wild-type Col (B and C). The RQ value corresponds to the average of three independent biological replicates. (A–C) Error bars represent the standard error between the biological replicates. The RQ value is represented on the top of each bar. *ACT11* was used as endogenous control for (A) and (B), *GAPC* for (C).

To investigate the effect of MSI1 on *MET1* expression, we used transgenic plants with a reduced level of MSI1 protein (*MSI1cs*) [36] since plants homozygote for the null *msi1* alleles cannot be obtained due to embryo lethality [28]. Quantitative PCR (Q-PCR) analyses showed a 5-fold increase of *MET1* expression in *MSI1cs* leaves in comparison to wild-type leaves (Figure 2A). We concluded that *MSI1* represses *MET1* expression. We performed bisulfite sequencing of the known methylated regions of the *FIS2* and *FWA* promoters to determine the level of CpG methylation in plants with reduced level of MSI1. Although most CpG sites in the two regions investigated are already methylated in the wild type, we could detect a modest increase of DNA methylation in leaves of *MSI1cs* plants (Figure S1).

In addition to RBR1, MSI1 participates in the Pc-G complex comprising MEA, FIS2, and FIE [28,29,37]. MSI1 is also an essential component of the *Arabidopsis* chromatin assembly factor-1 (CAF-1) complex with the two other proteins FASCIATA1 (FAS1) and FASCIATA2 (FAS2) [38]. The CAF1 complex is conserved in yeast, *Drosophila*, and mammals and is essential for the deposition of the heterodimer H3-H4 at the replication fork [39].

In order to investigate which *MSI1*-dependent pathway is responsible for the transcriptional control of *MET1*, we tested whether *MET1* expression was affected by loss of function of essential members of three distinct MSI1-dependent pathways involving *RBR1*, *FIE*, or *FAS1* [32]. We failed to obtain plantlets with a significant reduction of *RBR1* expression from *RBR1* RNA interference (RNAi) lines and thus have been unable to investigate the function of the Retinoblastoma pathway (unpublished data). Reduction of *FAS1* activity in plants homozygote for the null *fas1–1* allele [38] did not modify the level of *MET1* expression (Figure 2B). Similarly, the level of *MET1* expression was not affected by reduction of *FIE* activity in *FIE* cosuppressed plants (*FIEcs*) [40] (Figure 2B). We thus concluded that *MSI1*-mediated transcriptional repression of *MET1* is independent of the Pc-G and CAF-1 pathways. We thus hypothesized that *MET1* transcriptional control by *MSI1* may involve the Retinoblastoma pathway.

### MSI1 and RBR1 Bind to the *MET1* Promoter

**Figure 3:**
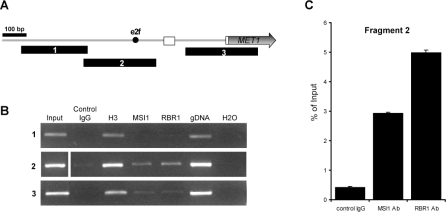
MSI1 and RBR1 Bind to the *MET1* Promoter (A) Schematic diagram of the *MET1* locus representing the fragments (black rectangles) analyzed by PCR after ChIP. White boxes represent the 5′ UTR, and the gray arrow corresponds to the first amino acid of exon 1 of *MET1*. A putative E2F binding site is represented by a black dot. (B) ChIP analysis using antibodies specific for MSI1 (MSI1 Ab) and RBR1 (RBR1 Ab) proteins. Nuclear extracts were prepared from wild-type Columbia buds after cross-linking. The first lane represents the input DNA. Control IgG is used as a negative control, while an antibody against histone 3 (H3) is used as a positive control. (C) Absolute quantification of the ChIP using Q-PCR for the fragment 2. Error bars represent the standard deviation of two independent PCR reactions.

To test whether MSI1 and RBR1 control directly *MET1* transcription by binding to the *MET1* promoter, we performed chromatin immunoprecipitation (ChIP) using antibodies against MSI1 (Figure S2) and RBR1 [41]. We focused our analysis on a 992-bp region of the *MET1* promoter spanning −869 bp to +123 bp relative to the predicted translation start site (Figure 3A). Chromatin from wild-type Columbia buds was immunoprecipitated with antibodies against MSI1 or RBR1. A significant enrichment in MSI1 and RBR1 binding was observed for the DNA fragment 2 spanning −608 bp to −303 bp relative to the start codon (Figure 3B and 3C). We conclude that MSI1 and RBR1 bind to the *MET1* genomic locus on a domain containing a putative E2F binding site (Figure 3A). These results strongly support the concerted action of MSI1 and RBR1 in regulating *MET1* expression.

### MSI1 and RBR1 Repress *MET1* Expression during Female Gametogenesis

**Figure 4:**
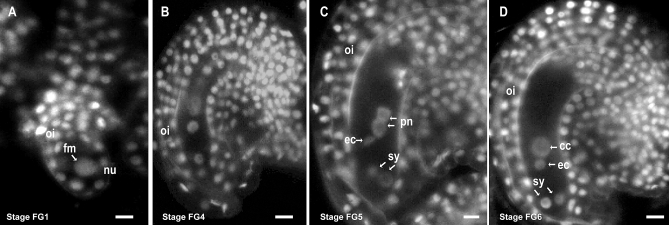
Expression the p*MSI1*-MSI1-mRFP1 Fusion Protein during Female Gametophyte Development Confocal images from transgenic plants expressing the MSI1-mRFP fusion protein under the control of its native promoter. MSI1-mRFP is expressed in nuclei of all the cells of the ovule integuments (oi). (A) Ovule with the functional megaspore (fm), where MSI1-mRFP accumulates in the nucleus. (B) Four-nucleate–stage (FG4) ovule. Only two of the four nuclei are visible. (C) Eight-nucleate–stage (FG8) ovule. (D) Mature female gametophyte. cc, central cell; ec, egg cell; nu, nucellus; pn, polar nuclei; sy, synergids. Scale bars represent 10 μm for (A) and 20 μm for (B–D).

**Figure 5:**
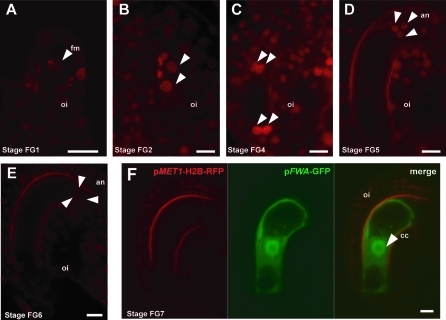
Expression the p*MET1*-H2B-RFP Reporter during Female Gametogenesis Confocal images from transgenic plants expressing the p*MET1*-H2B-RFP construct. (A) p*MET1*-H2B-RFP is expressed in the functional megaspore (fm) as well as in the ovule integument (oi). (B) Two-nucleate–stage (FG2) ovule. (C) Four-nucleate–stage (FG4) ovule. (D) Eight-nucleate–stage (FG8) ovule; p*MET1*-H2B-RFP expression is restricted to the antipodal nuclei (an). (E) Mature female gametophyte. (F) Colocalization of p*MET1*-H2B-RFP with *FWA*-GFP showing an absence of RFP in the mature central cell (cc), 1DAE. ec, egg cell; sy, synergids. Stages of female gametophyte development are indicated according to Christensen et al. [68]. Arrowheads point to nuclei. Scale bars represent 10 μm.

In plants with the genomic reporter construct p*MSI1:MSI1-mRFP*, we observed that MSI1-RFP was expressed throughout female gametogenesis (Figure 4) similar to RBR1 expression [27]. Coexpression of MSI1 and RBR1 during female gametogenesis suggested that *MET1* expression might be down-regulated in a specific manner in the female gametes. We obtained transgenic plants expressing the HISTONE2B fused to red fluorescent protein (RFP) under the control of the *MET1* promoter (p*MET1-H2B-RFP*). We determined the pattern of *MET1* expression during female gametogenesis by confocal microscopic observations of developing ovules from four independent transgenic lines. The H2B-RFP signal was observed in nuclei of the ovule integuments and in nuclei of the syncytial embryo sac from the megaspore till the four-nuclei stage (Figure 5A–5C). At the eight-nuclei stage, the signal can no longer be detected in the embryo sac apart from the three antipodal nuclei (Figure 5D). After cellularization of the eight nuclei, we could not observe any signal in the central cell, egg cell, and synergids, although some weak signal persisted in the three antipodals (Figure 5E). In the mature gametophyte, we could no longer detect any fluorescence from H2B-RFP in the central cell marked by p*FWA*-GFP expression (Figure 5F). As the result of our observations, we concluded that *MET1* is transcriptionally repressed in the central cell. Taking into account the expected H2B-RFP retention in nuclei after nuclear division, we estimated that *pMET1* activity is down-regulated at least from the four-nuclei stage of the female gametophyte.

**Figure 6:**
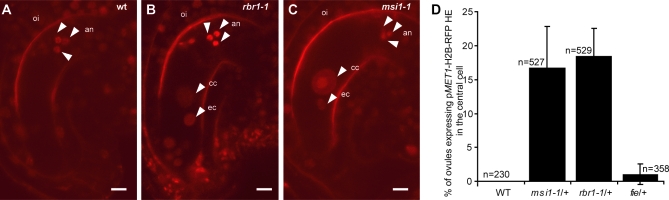
Control of *MET1* Expression by *MSI1* and *RBR1* (A) p*MET1*-H2B-RFP in wild-type mature ovules, the expression is restricted to the antipodal cells (an) and ovule integuments (oi). (B) p*MET1*-H2B-RFP in rbr1–1 ovules, ectopic RFP expression is observed in the central cell (cc) and egg cell (ec). (C) p*MET1*-H2B-RFP in msi1–1 ovule, ectopic RFP expression is observed in the central cell (cc) and egg cell (ec). Scale bar represent 10 μm. (D) Percentage of ovules expressing p*MET1*:H2B-RFP in the central cell in wild type (WT), *rbr1–1/+*, *msi1–1/+*, and *fie/+* plants. Arrowheads point to nuclei. Error bars represent the standard deviation. The *n* number is represented on the top of each bar.

In order to test the action of MSI1 on MET1 during female gametogenesis, we introduced the *MET1* transcriptional reporter in the *msi1* mutant background. In *msi1–1*/+ plants hemizygote for p*MET1*-H2B-RFP, the RFP signal was detected in about 25% of female gametophytes, the expected proportion of ovules inheriting both *msi1–1* and p*MET1*-MET1-RFP (Figure 6C and 6D). This result suggested the down-regulation of *MET1* expression by MSI1 in female gametophytes. We also observed an increased level of *MET1* transcripts in ovules from *msi1*/+ plants (Figure 2C), confirming that *MSI1* inhibits *MET1* expression in the female gametophyte. We did not detect any change of *pMET1* activity in the female gametophyte of the *fie*/+ mutant (Figure 6D), showing that *MET1* down-regulation in the female gametophytes is independent of *FIE*. However, we did observed an increased level of *MET1* transcripts in ovules from *fie*/+ plants (Figure 2C). Increased cell division activity in *fie* ovule integuments [27] could be responsible for the global increased *MET1* expression linked to its cell cycle dependence. To test whether *RBR1* was also required for the down-regulation of *MET1* in the female gametophyte, we studied *pMET1*-H2B-RFP expression in *rbr1* mutant. We observed ectopic p*MET1*-H2B-RFP expression in ovules from *rbr1–1*/+ plants (Figure 6B). RFP signal was observed in about 25% of female gametophytes from *rbr1*/+ plants hemizygous for the *pMET1-H2B-RFP* reporter (Figure 6D). We also directly observed increased *MET1* expression in *rbr1/+* ovules (Figure 2C) compared to wild type, which cannot result from integument cell proliferation because integument cells surrounding *rbr1* embryo sacs do not overproliferate in absence of fertilization [27]. We concluded that the Retinoblastoma pathway involving RBR1 and MSI1 down-regulates *MET1* expression during female gametogenesis.

### MSI1 and RBR1 Are Required for the Expression of *FIS2*

**Figure 7:**
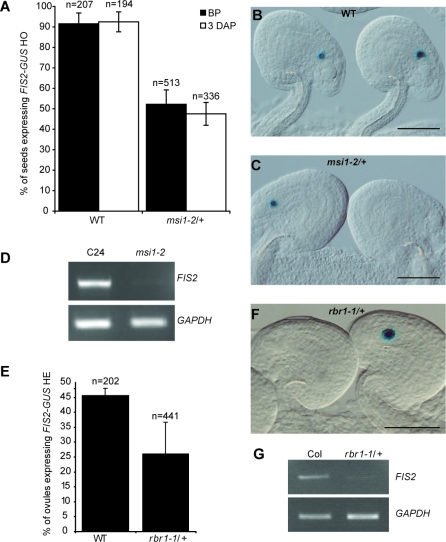
Control of *FIS2* Expression by *MSI1* and *RBR1* (A) Percentage of ovules/seeds expressing GUS in plants homozygous (HO) for *FIS2-GUS* in wild-type (WT) and *msi1–2/+* backgrounds. The percentage of ovules/seeds is represented before pollination (BP) and 3 d after pollination (3DAP). Error bars represent the standard deviation. The *n* number is represented on the top of each bar. (B and C) Photography illustrating *FIS2-GUS* expression in the central cell of wild-type (B) and *msi1–2/+* (C) ovules. Before pollination, it is not possible to distinguish on morphological bases the gametes carrying the wild-type or the *msi1–2* allele. Scale bars represent 50 μm. (D) RT-PCR on RNAs from seeds that inherited msi1–2 maternally selected on the basis of the overexpression of the fluorescent marker KS117 [28] (5 DAP). *GAPDH* is used as a control. (E) Percentage of ovules expressing GUS in plants hemizygous (HE) for *FIS2-GUS* in wild-type and *rbr1–1/+* backgrounds. Error bars represent the standard deviation. The *n* number is represented on the top of each bar. (F) *FIS2-GUS* expression in the central cell of *rbr1–1/+* ovules before pollination. Before pollination, it is not possible to distinguish on morphological bases the gametes carrying the wild-type or the *rbr1–1* allele. Scale bars represent 50 μm. (G) RT-PCR on RNAs from *rbr1–1*–selected ovules showing no *FIS2* expression in comparison to wild-type ovules. Selection of *rbr1–1* ovules was based on the lack of fertilization and seed development at 3 DAP in contrast to the wild-type ovules that were fertilized. *GAPDH* is used as a control.

In order to investigate the role of MSI1 and RBR1 on the transcription of imprinted genes silenced by MET1, we monitored the expression of the transcriptional reporter *FIS2-GUS* in a *msi1–2*/+ mutant background. Plants heterozygous for the null allele *msi1–2* produce 50% of *msi1–2* female gametes, which are deficient for MSI1 function [28,29]. We observed that only half of the ovules produced by *msi1–2*/+; *FIS2-GUS*/*FIS2-GUS* plants expressed the *FIS2-GUS* marker as compared to *FIS2-GUS* expression in the wild-type background (Figure 7A–7C), which correlated with the inheritance of *msi1–2* in 50% of the central cell (χ^2^ = 1.2, *p* > 0.27). The female gametogenesis is not perturbed by *msi1–2* [28,29], and the defects observed in *FIS2* expression are likely directly caused by the loss of *MSI1* function.

*FIS2-GUS* was also expressed in half of the developing seeds after fertilization (Figure 7A), Accordingly, *FIS2* transcripts were not detected by reverse-transcription PCR (RT-PCR) in isolated *msi1–2* mutant seeds selected on the basis of overexpression of the marker KS117 [28] (Figure 7D). MSI1 is expressed from both parental alleles in the endosperm [28]. If the wild-type *MSI1* allele paternally provided could rescue the inactive maternal allele of *FIS2* delivered by *msi1* ovules, we would expect 25% of ovules with *FIS2-GUS* expression. However, the proportion of seed without *FIS2* expression was 50%. This result indicates that the inactivated status of *FIS2* is inherited from the *msi1* ovule in the endosperm, supporting further a role of MSI1 in the imprinted status of *FIS2*. Similar results were obtained using the *msi1–1* allele (Figure S5). We concluded that *MSI1* is necessary for *FIS2* expression in the central cell and that the loss of *MSI1* in the central cell prevents the expression of *FIS2* in the endosperm.

To test whether the two *MSI1*-containing complexes, Pc-G and CAF1, are responsible for the repression of *FIS2* expression in absence of *MSI1*, we studied the effect of mutants in the genes *MEA*, *FIE*, and *FAS1*. We did not observe any loss of *FIS2-GUS* expression in ovules nor in endosperm of *mea-6*/+, *fie-11*/+, and *fas1–1*/+ mutants (Figure S3). Thus, activation of *FIS2* expression by *MSI1* is independent from the Pc-G and CAF-1 activities.

Altogether, the in vivo interaction between RBR1 and MSI1, their coexpression in the central cell, and their concerted regulation of *MET1* transcription, suggested a common requirement of *MSI1* and *RBR1* in the regulation of *FIS2* expression. Plants heterozygous for *rbr1–1*/+ loss-of-function allele showed a 50% reduction of ovules expressing the *FIS2-GUS* reporter in comparison to the wild-type background (Figure 7E and 7F). This reduction was correlated with the inheritance of *rbr1* in half of the ovules (χ^2^ = 0.27, *p* > 0.6). A dramatic reduction of *FIS2* expression was also detected by RT-PCR in *rbr1–1* mutant ovules (Figure 7G). We thus concluded that *RBR1* is necessary for *FIS2* expression in the central cell.

Since *FIS2* expression also depends on *DME* [12], we tested whether the loss of *FIS2* expression could be attributed to the loss of *DME* expression in response to the *MSI1/RBR1* pathway. *DME* and *MSI1* were still expressed in *rbr1* ovules, and conversely, the expression of *MSI1* and *RBR1* was not reduced in *dme* mutant buds (Figure S4). Our results suggest that the transcriptional controls of *DME* and *MSI1*/*RBR1* are mutually independent from each other.

### RBR1 and MSI1 Are Required for *FWA* Expression

**Figure 8:**
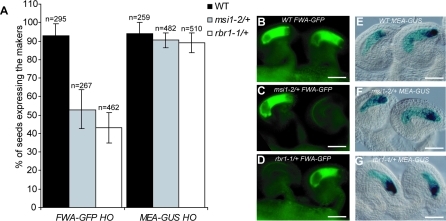
*RBR1* and *MSI1* Control Maternal *FWA* Expression but Not Maternal *MEA* Expression (A) Percentage of ovules expressing GFP or GUS activity from plants homozygous for *FWA-GFP* and *MEA-GUS* in wild-type (black bars), *msi1–2/+* (grey bars), and *rbr1–1/+* (white bars) backgrounds. Error bars represent the standard deviation. The *n* number is represented on the top of each bar. (B–D) *FWA-GFP* fluorescence in the central cell of wild-type (B), *msi1–2/+* (C), and *rbr1–1/+* (D) ovules. (E–G) *MEA-GUS* staining in the central cell of WT (E), *msi1–2/+* (F), and *rbr1–1/+* (G) ovules. Scale bars represent 50 μm.

The expression pattern of *FIS2* is similar to the expression patterns of the other MET1-dependent, maternally expressed imprinted gene, *FWA* [11,12]. We tested the effect of *rbr1* and *msi1* mutations on the expression of *FWA* using the transcriptional reporter *FWA-GFP* [11]. Only half of the ovules from *msi1–2*/+; *FWA-GFP/FWA-GFP* and from *rbr1–1*/+; *FWA-GFP*/*FWA-GFP* plants showed GFP expression in comparison to the expression of *FWA-GFP* in all wild-type ovules (Figure 8A–8D). The proportions of ovules that expressed *FWA-GFP* were in agreement with a gametophytic reduction of *FWA-GFP* expression by *msi1* (χ^2^ = 1.08, *p* > 0.3) and by *rbr1* (χ^2^ = 8.86, *p* > 0.003) in the mutant central cell. The repressed state of *FWA-GFP* also persisted in the endosperm of fertilized seeds from *msi1–2*/+; *FWA-GFP/FWA-GFP plants* (Figure S6). Similar results were obtained using the *msi1–1* allele (Figure S5). Hence, both *MSI1* and *RBR1* are required for *FWA* and *FIS2* expression.

### Activation of *FIS2* and *FWA* by MSI1 Is Mediated by the DNA Methyltransferase MET1

**Figure 9:**
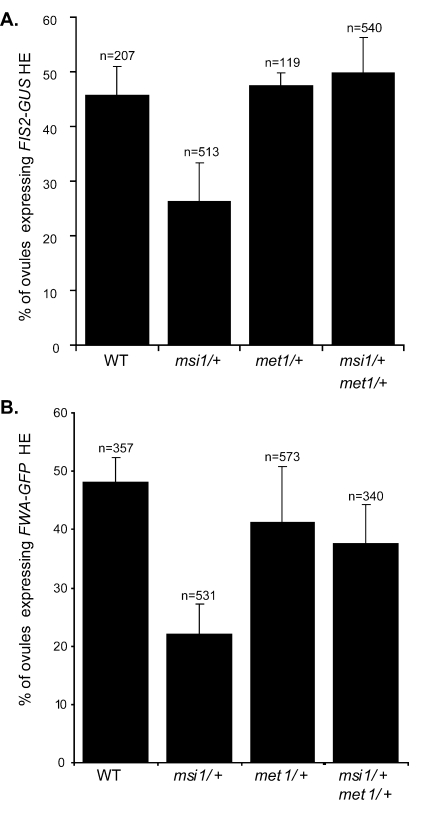
*MSI1* Activation of *FIS2* and *FWA* Is Mediated through *MET1* Percentage of ovules expressing GFP or GUS activity from plants hemizygous (HE) for *FIS2-GUS* (A) and *FWA-GFP* (B) constructs in wild-type, *msi1–2/+*, *met1–3/+*, and *msi1–3/+ met1–3/+* backgrounds. Error bars represent the standard deviation. The *n* number is represented on the top of each bar.

In order to investigate the genetic relationship between the activities of *MET1* and *MSI1*, we constructed a double-mutant plant of *msi1*/+; *met1*/+ carrying either *FIS2-GUS* or *FWA-GFP* reporters. In this experiment, we have used different *msi1* null alleles (described in Materials and Methods). *MSI1* and *MET1* are located only 3.6 Mb apart from each other on chromosome 5, which is estimated as 18 cM of linkage units. Considering the recombination frequency between the two genes, we expected that *msi1*/+; *met1*/+ plants would produce 41% of ovules carrying both the *msi1* mutation and the *met1* null mutation. If the effect of *msi1* and *rbr1* mutations on *FIS2* and *FWA* expression was mediated by a repression of *MET1*, we would have expected to observe 45.5% of ovules expressing *FIS2* or *FWA* in *msi1*/+; *met1*/+ plants (Table S1). We observed that 49.9% of the ovules of *msi1*/+; *met1–3*/+; *FIS2-GUS*/+ plants expressed the *FIS2-GUS* marker in comparison to 25.5% in the *msi1*/+; *FIS2-GUS*/+ background (Figure 9A). Thus the inheritance of *met1* mutation rescues the repression of *FIS2-GUS* expression by *msi1* (χ^2^ = 4.5, *p* > 0.03). We observed that 37.6% of the ovules of *msi1*/+; *met1*/+; *FWA-GFP*/+ plants expressed *FWA-GFP* in comparison to 22% in the *msi1*/+ background (Figure 9B). This indicated that the *met1* mutation antagonized the transcriptional repression of *FWA-GFP* by *msi1* (χ^2^ = 9.77, *p* > 0.01). Our results suggest that *MSI1* and *RBR1* antagonize the repressive action of *MET1*, which regulates the expression of *FIS2* and *FWA*. We conclude that *MET1* acts downstream of the *RBR1/MSI1* pathway, which is in agreement with our demonstration of the repression of *MET1* transcription in the central cell by the *RBR1/MSI1* pathway.

### RBR1 and MSI1 Are Not Required for *MEA* Expression

Silencing of *MEA* depends on H3K27 tri-methylation by Pc-G complexes [16,17], whereas silencing of *FIS2* and *FWA* is caused by DNA methylation by the maintenance methyltransferase *MET1* [12]. However, *MEA* activation in the central cell depends on DME action antagonized by MET1 [42]. Thus it remained to be tested whether *MET1* repression by RBR1 and MSI1 could have a direct impact on *MEA* expression in the central cell. In contrast to the transcriptional repression of *FIS2* and *FWA* mediated by *msi1* and *rbr1* mutants, we could not detect any impact of *msi1–2* or *rbr1–1* on the expression of the *MEA-GUS* reporter (Figures 8A, 8E–8G, and S6). Similar results were obtained using the *msi1–1* allele and the reporter *MEA-YFP* that encodes a fusion protein that complements *mea* (Figure S5). We thus concluded that *MEA* expression in the central cell is not regulated by *MSI1* and *RBR1*.

## Discussion

### Conservation of the Transcriptional Repression of MET1 by the Retinoblastoma Pathway

We identify a site in the putative promoter of *MET1* where MSI1 and RBR1 associate. In addition, we show that *MET1* expression is repressed by *MSI1*. This repression does not depend on the MSI1 association to the complexes Pc-G and CAF1, but depends on RBR1. Our results thus support a repression of *MET1* transcription by the MSI1/RBR1 complex. We have found one putative E2F binding site (ATTGCCGC) situated −387 bp from the predicted translation start site of the *MET1* promoter. Accordingly, *MET1* expression is strongly increased in plants overexpressing the *Arabidopsis* E2Fa and DPa proteins, suggesting that *MET1* is an E2F target gene [43,44] and *MET1* is expressed during the S phase of the cell cycle [45,46]. We thus propose that the activation of *MET1* transcription during the S phase requires the release of the sequestration of E2F by RBR1 at the G1/S cell cycle checkpoint. The RBR1/MSI1 complex would prevent E2F from activating the transcription of *MET1*. In human cells, the transcription of the human homolog of *MET1*, *Dnmt1* is also repressed by the Rb/E2F pathway [24,25]. Hence maintenance of DNA methylation would be coordinated with the S phase by an E2F control, and this mechanism is likely conserved in Eukaryotes.

### Repression of MET1 Is Essential for Activation of Imprinted Genes during Female Gametogenesis

MSI1 and RBR1 are expressed throughout female gametogenesis, and close inspection of *MET1* expression during female gametogenesis showed that *MET1* is specifically repressed during female gametogenesis by the Retinoblastoma pathway. The reduced expression of *MET1* in female gametes could explain why the inheritance of *met1* by the female gamete has no effect on endosperm and seed development [47]. In contrast, *MET1* is expressed in male gametes (Figure S7), and the inheritance of *met1* by the male gametes causes a strong reduction of endosperm and seed size [47,48] presumably as a result of the ectopic activation of the paternal allele of imprinted genes regulated like *FWA* and *FIS2*.

**Figure 10:**
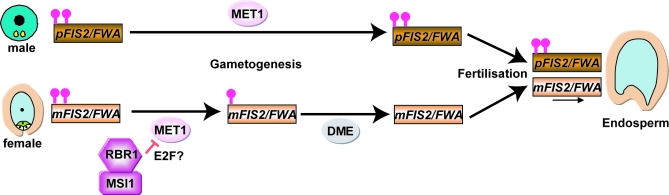
Model for MSI1/RBR1 Regulation of FIS2 and FWA Maternal Expression The MSI1/RBR1 complex represses the expression of *MET1* during female gametogenesis. As a result, the silencing DNA methylation marks (pink lollipops) are gradually lost during the female gamete nuclei divisions. In the central cell, DME removes the residual marks on imprinted genes such as *FIS2* and *FWA*, resulting in their transcriptional activation. The active status is conserved on the maternal allele during endosperm development. During male gametogenesis, *MET1* is expressed (Figure S7) and maintains the repression on the *FIS2* and *FWA* paternal allele. The paternal copy remains silent during endosperm development.

Our results indicate that the RBR1/MSI1 pathway activates the maternal expression of *FIS2* and *FWA* via the transcriptional repression of *MET1* in the central cell. The imprinted genes *FIS2* and *FWA* are repressed throughout the vegetative stage by MET1-dependent DNA methylation on their promoters [11,12]. At the end of female gametogenesis, DNA methylation is removed in the central cell by DME [11,16,19] leading to transcriptional activation. The demethylated maternal allele remains active in the endosperm. DME has a high affinity for hemimethylated DNA and causes excision of methylated cytosine residues followed by repair of single-strand break and incorporation of a nonmethylated cytosine residue [16,20]. However, we showed that DME activity is not sufficient to account for the activation of *FIS2* [12]. We propose that in addition to DME, the low level of MET1 activity during female gametogenesis causes a gradual dilution of the DNA methylation marks after each cycle of DNA replication (Figure 10). Following the third cycle of syncytial division, the central cell and the egg cell become isolated from each other. In the egg cell, the remaining marks are sufficient to maintain *FIS2* and *FWA* silenced before fertilization and after fertilization in the embryo. In contrast, the DNA glycosylase DME is expressed in the central cell [19] and previous enrichment in hemimethylated DNA at the *FIS2* and *FWA* loci would prime DME to remove the remaining marks leading to activation of *FIS2* and *FWA*. If the Retinoblastoma pathway does not function during female gametogenesis, DNA methylation is retained causing the absence of activation of *FIS2* and *FWA*, thus preventing their maternal expression in the endosperm.

Although *FWA* and *FIS2* repression by *MET1* appear similar, *FWA* expression is completely dependent on DME, whereas *FIS2* expression is still expressed even in absence of DME activity. Similarly, loss of MET1 in vegetative tissues causes *FWA* ectopic expression, but *FIS2* remains silenced. This suggests that at least another transcriptional activator control *FIS2* expression in the endosperm lineage.

### *MEA* Imprinting Is Not Primarily Controlled by MET1

Previous studies have shown that *MEA* expression is regulated by MET1 and DME [16,42]. However, it remained unclear whether MET1 and DME regulate *MEA* imprinting directly. Genome-wide array of DNA methylation and H3K27 trimethylation have shown that the *MEA* locus is covered with H3K27 trimethylation but devoid of DNA methylation. DNA methylation can be found in the *MEA* locus only at repeats at the 3′ end of the gene, the *MEA* ISR domain [49–51]. However, a *MEA* reporter construct that does not contain the *MEA* ISR domain displays imprinted expression and complements the *mea* mutant phenotype [37] strongly suggesting that the *MEA* ISR is not involved in the regulation of *MEA* expression. Furthermore, the direct loss of DNA methylation in *met1* pollen does not cause activation of the *MEA* paternal allele in endosperm [12], whereas *MEA* is activated directly by the loss of H3K27 trimethylation in mutants for Pc-G activity in both vegetative and reproductive tissues [16,17]. We do not observe any impact of the ectopic expression of MET1 by *msi1* and *rbr1* on *MEA* expression. Hence, we can conclude that *MEA* expression is not directly controlled by MET1. Rather, the direct silencing of *MEA* by H3K27 methylation implies that trimethylated H3K27 must be removed from the *MEA* locus during female gametogenesis to obtain transcriptional activation. It is thus possible that MET1 and DME are required directly or indirectly for the activation of a pathway that removes the H3K27 trimethylation mark from *MEA* leading to its activation. Alternatively, maternal *MEA* expression may require a transcriptional activator that is itself directly controlled by DNA methylation and DME activity.

### A Genome-Wide Demethylation during Female Gametogenesis?

It is not clear whether DNA demethylation during female gametogenesis affects only a discrete number of loci as a result of still-unknown targeting mechanisms. Alternatively, the DNA demethylation regulated by the Retinoblastoma pathway could affect the entire genome leading to a reduction of the constitutive heterochromatin fraction in the central cell, which could be inherited in the endosperm. A global decrease of maternal DNA methylation in the endosperm was suggested to occur in maize based on the analysis of methylation-sensitive amplified polymorphism [52] and in *Arabidopsis* based on the reduction of the heterochromatin fraction [53]. A global demethylation is not expected to affect significantly DNA of the egg cell since it has been clearly established that transgenes silenced by DNA methylation remain silenced from one generation to the next [54].

### Potential Conservation of Regulation of Imprinting by the Retinoblastoma Pathway

Our results provide evidence for a direct link between the Retinoblastoma pathway and genomic imprinting. The regulation of the CpG maintenance methyltransferase by the Retinoblastoma pathway is conserved between plants and mammals. Although there has been no direct evidence in mammals for a role of the Retinoblastoma protein itself in genomic imprinting, in a human cell line, the up-regulation of the expression of the DNA methyltransferase *Dnmt1* caused by misregulation of pRb was correlated with an increase of CpG methylation of the paternally expressed imprinted gene *Peg3* [25]. The loss of function of pRb during mouse embryonic development results in embryonic lethality. However, this lethality can be partially rescued by providing mutant embryo with a wild-type placenta, demonstrating a crucial role of pRb during placenta development. pRb deficiency in the placenta results in a decrease of nutrient transport from the mother to the embryo [55,56]. The identification of two Retinoblastoma binding proteins (Rbbp1/Arid4a and Rbbp1l1/Arid4b) that are required for the correct epigenetic marks and imprinting status at the PWS/AS domain provides further evidence for a potential role of pRb on imprinting during placenta development [57]. The data above indicate that the impact of the Retinoblastoma pathway on parental genomic imprinting in plants, that we have demonstrated may also exist in mammals and would thus provide further evidence for a convergence of imprinting between flowering plants and mammals.

## Materials and Methods

### Plant materials and growth conditions.

Mutants *fie-11*, *mea-6*, and *msi1–2* were previously characterized in our laboratory. *rbr1–1*/+ (SALK_012270) and *rbr1–2*/+ (SALK_002946) were previously described by Ebel et al. [58]. The *msi1–1*/+ mutant line [59] and the *MSI1cs* line were kindly provided by Lars Hennig [36]. *FIEcs* plants were previously characterized [40]. *fas1–1* was described in Kaya et al. [38]. The met1–3 line was provided by J. Paszkowski [54]. The MEA-YFP reporter line was kindly provided by R. Yadegari [37]. The KS117 line was identified after a screen in the Jim Haseloff's enhancer trap GFP line collection [60]. The transgenic reporter lines *FIS2* promoter-*GUS* and *MEA* promoter*-GUS* fusions constructs were kindly provided by A. Chaudhury [18]. The *FWA* promoter-*GFP* was kindly provided by T. Kinoshita [11]. The p*MSI1:*MSI1-mRFP1 locus fusion was generated in our laboratory [61]

After 3 d at 4 °C in the dark, seeds were germinated and grown on soil. Plants were maintained in a growth chamber under long days at 20 °C (16-h light/8-h night).

### RNA extraction, cDNA preparation, and RT-PCR.

Sample tissues were collected from *Arabidopsis* plants and frozen in liquid nitrogen. As only *msi1–2*/+ plants survive, we selected *msi1–2* mutant seeds characterized by the overexpression of the KS117 marker at 5 d after pollination [28]. As only *rbr1–1*/+ plants survive, we selected *rbr1–1* mutant ovules using the size difference between undeveloped *rbr1–1* ovules and wild-type seeds 1 DAP before *rbr1* ovules degenerate (4 DAP) [27]. RNA extraction, DNase treatment, and reverse transcription were performed as described previously [12]. Primer sequences are listed in Table S2.

### Quantitative real-time RT-PCR.

Real-time PCR assays were performed using Power SYBR Green PCR Master Mix (Applied Biosystems). One microliter of RT product was used to perform each PCR reaction. Amplification reaction was carried out using specific primers at a concentration of 0.5 mM in a 10-μl reaction. The *MET1*-specific primer pair was qPCR-MET1_Rev2 and qPCR-MET1_Fwd2 (Table S2).

The specificity of the amplification was determined by performing a dissociation curve analysis. The PCR reaction and quantitative measurements were achieved with 7900HT Fast Real-Time PCR System (Applied Biosystems). Thermal cycling parameters were 2 min at 50 °C, 10 min at 95 °C, and 50 cycles of 15 sec at 95 °C, 60 sec at 60 °C. Three technical replicates were done for each sample. The ΔCt was calculated using *ACT11* gene as endogenous control (Table S2). Relative quantitation (RQ) values were calculated using the 2^−ΔCt^ method (RQ = 2^−ΔCt^) [62]. Values given in Figure 2A represent the RQ average of five biological replicates for each point and three biological replicates (Figure 2B and 2C).

### Chromatin immunoprecipitation.

The ChIP experiment was performed as described previously [63] with minor modifications. Buds were ground with a pestle in liquid nitrogen and fixed with 1% formaldehyde for 10 min. The chromatin was precleared by incubating with protein A beads (Upstate) and IgY-beads (Aves Labs). The protein–DNA complexes were incubated overnight with 40 μl of MSI1 antiserum (Figure S2), 5 μl of purified RBR1 antibody [41], 10 μl of Rabbit Control IgG (Abcam, ab46540) or 20 μl of Histone H3 antibodies (Abcam, ab1791) and immunoprecipitated with protein A beads (Upstate) and IgY-beads (Aves Labs) respectively. After reverse cross-linking, proteinase K, and RNase treatment, the immunoprecipitated DNA was purified using a silica-gel membrane (Qiagen) and analyzed by PCR. PCR reactions were performed in 20 μl using HotStarTaq DNA Polymerase (Qiagen). Quantitative measurements of enrichment from the fragment 2 of *MET1* were performed using the absolute quantitation method achieved with 7900HT Fast Real-Time PCR System (Applied Biosystems). Relative enrichments were calculated as the percentage of the obtained values in immunoprecipitated and input fractions. Primer sequences are listed in Table S1.

### GST pull-down assay.

A full-length *MSI1* cDNA was cloned into the BamHI site of pGEX 4T-1 vector (Amersham Pharmacia) in frame to GST. *RBR1* was cloned in to the pCITE 3a (Novagen) as described [64]. Pull-down assays were performed as described previously [65]. A GST-MSI1 fusion protein was expressed in *Escherichia coli* BL21 cells and immobilized on glutathione agarose beads. Beads were incubated with radioactively labeled RBR1 protein followed by six consecutive washes with NETN buffer (100 mM NaCl, 1 mM EDTA, 20 mM Tris [pH 8.0], and 0.5% NP-40). Protein labeling was performed using a coupled transcription-translation system in a reticulocyte lysate system (Promega) according to the manufacturers instructions using radiolabeled [^35^S] methionine (Amersham-Pharmacia). Following SDS/PAGE, the translated RBR1 products appeared as a major band of 120 kDa, as expected. Beads were washed and resuspended in SDS–polyacrylamide gel sample buffer (60 mM Tris [pH 6.8], 2% SDS, 10% glycerol, 0.002% bromphenol blue, and 100 mM DTE). Sample were separated by electrophoresis on a 10% SDS–polyacrylamide gel, and transferred to a polyvinylidene difluoride (PVDF) Immobilion-P membrane (Millipore). Labeled RBR1 protein was detected by autoradiography. The GST protein was detected using anti-GST antibodies (Amersham Pharmacia Cat#27457701) diluted 1:1,000 (v:v).

### Analysis of protein–protein interaction in plants by bimolecular fluorescence complementation assay.

Protein–protein interactions in plants were examined by BiFC assay [31]. Equal concentrations of Agrobacterium tumefaciens strain GV3101/pMp90 containing plasmids of interest (Table S3) were transiently coexpressed in Nicotiana benthamiana leaves via leaf injection procedure. Following incubation at 25 °C for 24 h, samples were examined with Leica TCS-SL confocal laser scanning microscope with 20× and 63× water immersion objectives. Image analysis was performed with Zeiss AxioVision, Zeiss CLSM-5, and Adobe Photoshop 7.0 YFP was visualized by excitation with an argon laser at 514 nm. Emission was detected with spectral detector set between 525 nm and 570 nm. *RBR1* and *MSI1* full-length cDNA and three deletion constructs of *RBR1* were cloned into the SpeI site of pSY 735 and pSY 736 containing the N-terminal (YN), or C-terminal (YC) fragments of the YFP protein, respectively [31]. Negative controls with vectors bearing only YN or YC alone were carried out in every experiment to verify the specificity of the interactions. Each confocal section detects only a fraction of the nuclei in a given field of view, and not every cell expresses both constructs. This explains why only a fraction of cells show fluorescent nuclei in a given field of view when interaction takes place.

### Microscopy and statistics.

*FIS2-GUS*, *FWA-GFP*, *MEA-GUS*, and MEA:YFP expression were analyzed as previously described [12,17]. Developing seeds or pistils cleared with derivative of Hoyer's medium were observed with differential interference contrast (DIC) optics and with 20× PlanApo objective (DM6000 B, Leica). Images were acquired with a DXM1200F digital camera (Nikon) and processed using Metamorph (version 6.2, Universal Imaging Corp). In Figures 7, 8, 9, S3, S5, and S6, the error bars represent the standard deviation calculated from the mean measured per silique containing, on average, 40 ovules. The *n* number is represented on the top of each bar and corresponds to the total number of ovules observed.

pMSI1-MSI1-RFP and pMET1-H2B-RFP fluorescence was imaged using laser scanning confocal microscopy (Zeiss Exciter) for mRPF1 with selective settings for RFP detection (excitation, 543 nm; emission, band-pass 560 to 615 nm).

### pMET1:H2B-mRFP1 plasmid construction and transformation.

The MET1 promoter was amplified by PCR using the KOD-plus-PCR kit (TOYOBO) and primer pair: R2met1-F1td and MET1-int66R (Table S2). The PCR fragment was then cloned into pENTR-D-TOPO (Invitrogen). The promoter was then fused to H2B-RFP in frame by recombination using Gateway technology (Invitrogen) into the pGreenII-GW-H2B-RFP destination vector [66]. The final vector (pGreenII-*pMET1*-H2B-RFP) contains 947 bp upstream of the translation start site until the first 66 amino acids of MET1 in frame with H2B-RFP. Wild-type Columbia were transformed using the *Agrobacterium*-mediated floral dip method [67], and transgenic lines were selected on kanamycin. The presence of the transgenes was confirmed using PCR. Thirteen transgenic lines showed consistent patterns of transgene expression. Three transgenic lines were used for the detailed observation of the expression pattern using confocal microscopy.

## Supporting Information

Figure S1 Comparison of CpG DNA Methylation between Wild-Type and *MSI1cs* Plants In order to know whether increased *MET1* expression in MSI1cs leaves also leads to an increase in the amount of CpG methylation, we performed bisulfite sequencing of the methylated region of the *FIS2* and *FWA* promoters.
(A) Analysis of CpG methylation of *FIS2* promoter region using bisulfite sequencing. Twelve clones were sequenced for wild-type Col, and 11 clones were sequenced for MSI1cs.
(B) Analysis of CpG methylation of *FWA* promoter region using bisulfite sequencing. Eleven clones were sequenced for wild-type Col, and eight clones were sequenced for MSI1cs.
In comparison to wild-type levels, we observed in MSI1cs plants a slight increase in the methylation of the *FIS2* promoter, but did not see any significant modification of the methylation level in the *FWA* promoter. Several factors may explain this limited effect. Increased level of *MET1* mRNA may not result in an increased MET1 activity or the increase of MET1 activity in vegetative tissues is not sufficient to be effective. Alternatively, the targets studies may not be responsive to increased MET1 activity in vegetative tissues. The most likely hypothesis is that *FIS2* and *FWA* methylated regions are already highly methylated in wild-type leaves, which prevents the observation of an increase in MSI1cs.
Methods: bisulfite-sequencing analysis was carried out with the EZ DNA Methylation-Gold Kit (Zymo Research). The treated DNA was cleaned up in accordance with the manufacturer's instructions and used for subsequent PCR. After PCR, the products were cloned in pGEM-Teasy (Promega), and individual clones were sequenced with SP6 and T7 primers. The sequences were then aligned with AlignX (Invitrogen), and the methylation level was analyzed using BiQ Analyzer (http://biq-analyzer.bioinf.mpi-sb.mpg.de). Primers used for bisulfite-sequencing analyses are listed in Table S1. The *ASA1* gene was used as a positive control of bisulfite chemical reaction (Kinoshita et al. [11]).
(928 KB EPS)

Figure S2 Specificity of the Antiserum against MSI1 Antibodies against the C-terminal region of MSI1 were obtained from immunization of rabbits with the peptide MGKDEEEMRGEIEERLINE (Invitrogen). A western blot was performed using serum against protein extracts from 10-d-old seedlings from *Arabidopsis*, wild-type Columbia, and a transgenic Columbia line carrying the gene encoding the MSI1-RFP protein fusion under the control of the MSI1 promoter (Figure 4). A major band was detected by the antibody in the wild-type background around 55 kDa, i.e., slightly above the predicted molecular weight of MSI1 (48.2 kDa). A minor band was detected at 68 kDa. In the extract of the MSI1-RFP line, an additional band was detected at 80 kDa. The molecular weight of RFP is 27 kDa, and the predicted protein fusion weight of 82 kDa corresponds to the size observed, indicating that the major protein detected by the antibody is MSI1. The intensity of the major band in the wild type was reduced, indicated a potential cosuppression effect of the expression of an additional copy of MSI1.
(2.37 MB EPS)

Figure S3 *FIS2-GUS* Expression Is Not Affected by PcG and CAF-1 Mutation (A) Percentage of ovules or seeds expressing *FIS2-GUS* (homozygote for the marker [HO]) in wild type (WT) and in Polycomb mutants *mea-6*/+ and *fie-11*/+ before pollination (BP) and 2.5 d after pollination (2.5 DAP).
(B) Percentage of seeds expressing *FIS2-GUS* (hemizygote for the marker, HE) in wild type (WT) and in CAF-1 mutant *fas1–1*/+ before pollination.
Error bars represent the standard deviation. The *n* number is represented on the top of each bar.
(561 KB EPS)

Figure S4 RBR1/MSI1 Pathway Is Independent of DME (A) RT-PCR on RNAs from *rbr1–1*–selected ovules showed no reduction of *DME* and *MSI1* in *rbr1* ovules in comparison to wild-type Col accession. *GAPDH* is used as a control.
(B) RT-PCR on RNAs from *dme-4* buds showing no reduction of *RBR1* and *MSI1* in *dme-4* buds in comparison to wild-type C24 accession. *GAPDH* is used as a control.
(440 KB EPS)

Figure S5 *FWA*-GFP, *FIS2*-GUS, and *pMEA*-MEA-YFP Marker in a *msi1–1*/+ Mutant Background Percentage of seeds expressing *FWA-GFP* hemizygote (A), *FIS2*-GUS hemizygote (B), and *pMEA*-MEA-YFP homozygote (C) in wild type and *msi1–1*/+ in mature ovules before pollination (BP). Error bars represent the standard deviation. The *n* number is represented on the top of each bar.
(579 KB EPS)

Figure S6 MSI1 Is Required for *FWA-GFP* Expression in the Endosperm Percentage of seeds expressing *MEA-GUS* and *FWA-GFP* in wild-type (black bar) and *msi1–2*/+ (white bar) in seeds at 2.5 DAP. Error bars represent the standard deviation. The *n* number is represented on the top of each bar.
(395 KB EPS)

Figure S7 Expression the pMET1:H2B-RFP Marker during Male Gametogenesis Epifluorescence images from transgenic plants expressing the p*MET1*:H2B-RFP fusion protein costained with DAPI.
(A) p*MET1*:H2B-RFP is expressed in the microspore (M).
(B) At the bicellular stage, p*MET1*:H2B-RFP expression is restricted to the generative cell (G) and is absent from the vegetative cell (V).
(C) At the tricellular stage, p*MET1*:H2B-RFP is expressed in the two sperm cells (S). Scale bars represent 10 μm.
(3.57 KB EPS)

Table S1 Expected Percentage of Ovules Expressing the Marker in a *met1*/+;*msi1*/+ Plant (28 KB DOC)

Table S2 List of Primers (54 KB DOC)

Table S3 Vectors Using YFP in BiFC Analyses (37 KB DOC)

## Abbreviations

BiFC: bimolecular fluorescence complementation
CAF-1: chromatin assembly factor-1
ChIP: chromatin immunoprecipitation
DME: DEMETER
FAS1: FASCIATA1
GST: glutathione S-transferase
HDAC: histone deacetylase
H3K27: histone 3 lysine 27
MET1: METHYLTRANSFERASE 1
MSI1: MULTICOPYSUPPRESSOR OF IRA1
Pc-G: polycomb group
pRb: Retinoblastoma protein
Q-PCR: quantitative PCR
RBR1: RETINOBLASTOMA RELATED 1
RFP: red fluorescent protein
RQ: relative quantitation values
RT-PCR: reverse-transcription PCR
YC: C-terminal part of the yellow fluorescent protein
YFP: yellow fluorescent protein
YN: N-terminal part of the yellow fluorescent protein
