# Supplementary material for: Retinoblastoma and Its Binding Partner MSI1 Control Imprinting in Arabidopsis
Source: PLoS Biol. 2008 Aug 12;6(8):e194. doi: 10.1371/journal.pbio.0060194 (PMC2504488; doi:10.1371/journal.pbio.0060194)
Supplement: Supplementary file 1 [file pbio.0060194.st001.doc]

**Table S1.** Expected percentage of ovules expressing the marker in a *met1*/+;*msi1*/+ plant.

| Ovule genotypes | wt | *met1* | *msi* | *msi*;*met1* |
| --- | --- | --- | --- | --- |
| *msi* and *met1* linked by  18 cM | 41 % | 9 % | 9 % | 41 % |
| Marker heterozygote | 20.5 % | 4.5 % | 4.5 % | 20.5 % |
| If *met1* suppresses *msi1* effect on marker expression | marker expressed | marker expressed | marker not expressed | marker expressed |
|  | 45.5 % of ovules should express the marker | | | |
| If *met1* does not suppress *msi1* effect on marker expression | marker expressed | marker expressed | marker not expressed | marker not expressed |
|  | 25 % of ovules should express the marker | | | |
