# Supplementary material for: Retinoblastoma and Its Binding Partner MSI1 Control Imprinting in Arabidopsis
Source: PLoS Biol. 2008 Aug 12;6(8):e194. doi: 10.1371/journal.pbio.0060194 (PMC2504488; doi:10.1371/journal.pbio.0060194)
Supplement: Supplementary file 2 [file pbio.0060194.st002.doc]

**Table S2.** List of primers.

|  | genes | Primer name | Primer sequence |
| --- | --- | --- | --- |
| RT-PCR | FIS2 | Fis2-R5018 | CCTGCATTGTTTGGGAGTGATAGAA |
|  |  | Fis2-F3412 | GGATGATGTAGGAAATCCCCAATTGAGCCCTTTG |
|  | MET1 | MER-1 | GTTAAGCTCATTCATAGCCTTGC (Saze et al. 2003) |
|  |  | MEF-1 | GATTGTGTCTCTACTACAGAGGC (Saze et al. 2003) |
|  | GAPDH | GAPDH3’ | GTAGCCCCACTCGTTGTCGTA |
|  |  | GAPDH5’ | AGGGTGGTGCCAAGAAGGTTG |
| Q-PCR | MET1 | qPCR-MET1_Rev2 | CATTGATGAAGTCCACTTGAC |
|  |  | qPCR-MET1_Fwd2 | GTGATTCTTAGGGCTATAATGG |
|  | ACT11 | qPCR-Act11_Fwd1 | GAGATGATGCACCAAGAGCTGTA |
|  |  | qPCR-Act11_Rev1 | CATACCAACCATGACACCAGTGT |
|  | GAPC | qPCR-GAPC_Fwd2 | actcaatcactgctactcag |
|  |  | qPCR-GAPC_Rev2 | gtcaactttccgttaagagc |
| Bisulfite sequencing | FIS2 | FIS2Bis-F4 | TATAAATATGTTATATAGGTTT |
|  |  | FIS2Bis-R4 | CCTACATTTTAATAAAATATTAC |
|  | FWA | FWABis-F2 | TAYTAATATYAAAGAGTTATGG |
|  |  | FWABis-F3 | TTTGTGGGATATTGATAATTAG |
|  |  | FWABis-R2 | AATCTRATTRTCARTATCCCAC |
|  |  | FWABis-R3 | AAATACAACCARAATAAAATTC |
| ChIP | MET1 Frag 1 | R2met1-F13 | CGGCATCCTACCAATTTGAG |
|  |  | R2met1-R13 | CAGTCAACAGTCAAAGTATACGAG |
|  | MET1 Frag 2 | R2met1-F12 | TACTTTGACTGTTGACTGATGG |
|  |  | R2met1-R12 | GTGTGATTTGAGAGGAAGAG |
|  | MET1 Frag 3 | R2met1-F14 | TGATTGTTAAACCCTAGACTCGG |
|  |  | R2met1-R14 | CTTGAAACTGGTACACGCTG |
| pMET1 Cloning | R2met1-F1td | | CACCTCATGGTAAAATGTTAGTTC |
|  | MET1-int66R | | AAACTCTTCCTCCACAATCTG |
|  |  |  |  |
|  |  |  |  |
|  |  |  |  |
|  |  |  |  |

Reference:

Saze, H., Scheid O.M. and Paszkowski J.. 2003. Maintenance of CpG methylation is essential for epigenetic inheritance during plant gametogenesis. *Nat Genet* **34**: 65-69.
