# Supplementary material for: Retinoblastoma and Its Binding Partner MSI1 Control Imprinting in Arabidopsis
Source: PLoS Biol. 2008 Aug 12;6(8):e194. doi: 10.1371/journal.pbio.0060194 (PMC2504488; doi:10.1371/journal.pbio.0060194)
Supplement: Supplementary file 3 [file pbio.0060194.st003.doc]

**Table S3.** Vectors used YFP in BiFC analyses

| Plasmid Name | Description | Source |
| --- | --- | --- |
| pSY 736 | YN only (For negative control) | (Bracha-Drori et al. 2004) |
| pSY 735 | YC only (For negative control) | (Bracha-Drori et al. 2004) |
| pSY 736 AtMSI | YN-MSI1 | This study |
| pSY 735 AtMSI | YC-MSI1 | This study |
| pSY 736 AtRBR | YN-RB | This study |
| pSY 735 AtRBR | YC-RB | This study |
| pSY 736 AtRBR 1192-2676 | YN-RB  AB (aa: 398-892) | This study |
| pSY 735 AtRBR 1192-2676 | YC-RB  AB (aa: 398-892) | This study |
| pSY 736 AtRBR 1-1809 | YN-RB A (aa: 1-604) | This study |
| pSY 735 AtRBR 1-1809 | YC-RB A (aa: 1-604) | This study |
| pSY 736 AtRBR 2173-3042 | YN-RB B (aa: 725-1013) | This study |
| pSY 7385 AtRBR 2173-3042 | YC-RB B (aa: 725-1013) | This study |

Bracha-Drori, K., Shichrur K.,. Katz A, Oliva M., Angelovici R., Yalovsky S. and Ohad N. 2004. Detection of protein-protein interactions in plants using bimolecular fluorescence complementation*. Plant* **J** 40: 419-427.
